# Involvement of Periodontal Disease in the Pathogenesis and Exacerbation of Nonalcoholic Fatty Liver Disease/Nonalcoholic Steatohepatitis: A Review

**DOI:** 10.3390/nu15051269

**Published:** 2023-03-03

**Authors:** Takashi Kobayashi, Michihiro Iwaki, Asako Nogami, Yasushi Honda, Yuji Ogawa, Kento Imajo, Satoru Saito, Atsushi Nakajima, Masato Yoneda

**Affiliations:** 1 Department of Gastroenterology and Hepatology, Yokohama City University Graduate School of Medicine, 3-9 Fukuura, Kanazawa, Yokohama 236-0004, Japan; 2 National Hospital Organization Yokohama Medical Center, Gastroenterology Division, 3-60-2 Harajyuku, Yokohama 245-8575, Japan; 3 Department of Gastroenterology, Shin-Yurigaoka General Hospital, 255 Tsuko, Furusawa, Kawasaki 215-0026, Japan

**Keywords:** nonalcoholic fatty liver disease, nonalcoholic steatohepatitis, periodontitis, dysbiosis, multiple parallel hits, treatment, *P. gingivalis*

## Abstract

The increasing incidence of nonalcoholic fatty liver disease (NAFLD) and nonalcoholic steatohepatitis (NASH), along with global lifestyle changes, requires further in-depth research to elucidate the mechanisms and develop new treatment strategies. In addition, the number of patients with periodontal disease has increased recently, suggesting that periodontal disease is sometimes associated with systemic conditions. In this review, we summarize recent studies linking periodontal disease and NAFLD, the concept of the mouth–gut–liver axis, oral and intestinal microbiota, and liver disease. We suggest new research directions toward a detailed mechanistic understanding and novel targets for treatment and prevention. Forty years have passed since the concepts of NAFLD and NASH were first proposed. however, no effective prevention or treatment has been established. We also found that the pathogenesis of NAFLD/NASH is not limited to liver-related diseases but has been reported to be associated with various systemic diseases and an increasing number of causes of death. In addition, changes in the intestinal microbiota have been shown to be a risk factor for periodontal diseases, such as atherosclerosis, diabetes, rheumatoid arthritis, nonalcoholic fatty liver disease, and obesity.

## 1. Introduction

Nonalcoholic fatty liver disease (NAFLD) and nonalcoholic steatohepatitis (NASH) are the most common liver diseases. There has been a rapid increase in the number of patients worldwide with these diseases due to the rising prevalence of obesity and type 2 diabetes. The prevalence of NAFLD is 25.2%, with approximately 10–20% of these patients considered to have NASH. The prevalence is increasing yearly [1,2].

The multiple parallel hits hypothesis, which states that NAFLD/NASH is caused by the simultaneous action of many factors, such as obesity; diabetes including insulin resistance, dyslipidemia, and other factors of metabolic syndrome; disruption of the intestinal microflora; and cytokine abnormalities, has become the mainstream hypothesis [3,4]. 

Periodontal disease is a general term for a group of diseases characterized by inflammatory lesions in the periodontal tissues (gingiva, periodontal ligament, cementum, and alveolar bone). The cause of periodontal disease is dental plaque, a biofilm-like growth of oral bacteria. The inflammatory response to plaque bacteria causes the formation of deep periodontal pockets between the teeth and gingiva. Deep periodontal pockets provide a favorable environment for the growth of anaerobic periodontopathogenic bacteria, and persistent inflammation leads to resorption and destruction of the periodontal ligament and alveolar bone, supporting tissues of the teeth, and deep gingival epithelial growth, resulting in further periodontal pocket deepening. Among the hundreds of bacterial species found in deep periodontal pockets, some Gram-negative anaerobes, such as *P. gingivalis*, are thought to be pathogenic bacteria that contribute to the pathogenesis of the periodontal disease. When periodontitis is present in all teeth, the size of ulcers in periodontal pockets can reach 50 cm^2^ or more [5]. It is assumed that inflammatory cytokines and bacteria are hematogenously transmitted from ulcers in periodontal pockets to the whole body, resulting in changes in the intestinal microflora and involvement in various diseases, including type 2 diabetes [6,7,8,9]. Recent oral bacteriological and epidemiological studies have reported many effects of oral bacteriology, especially periodontal disease, on the whole body. Therefore, the concept of “periodontal medicine,” in which periodontal disease is involved in a bidirectional manner with systemic diseases, has been proposed [10].

NAFLD and NASH, liver diseases that develop from lifestyle-related diseases, have also been reported to be associated with periodontal disease. The relationship between periodontal disease and abnormal liver function in humans was first reported by Furta et al. in 2010 [11]. Later, the relationship between *Porphyromonas gingivalis* (*P. gingivalis*) and NAFLD was first reported by Yoneda et al. in 2012 [12], and has since attracted much attention. However, the increasing incidence of NAFLD and NASH, along with global lifestyle changes, requires further in-depth research to elucidate the mechanisms and develop new treatment strategies. Herein, we review the association between periodontal disease and NAFLD/NASH.

## 2. Epidemiology of NAFLD/NASH and Periodontal Disease

Epidemiological studies have reported significant associations between NAFLD and periodontal disease worldwide. In Korea, 4272 participants were investigated based on the Korea National Health and Nutrition Examination Survey. The fatty liver index (a surrogate index for fatty liver calculated from the body mass index, abdominal circumference, serum γ-glutamyl transpeptidase [γGTP] level, and serum triglyceride level) and periodontal-disease-related index (community periodontal index) were reported to be associated with periodontal disease [13]. In China, the number of missing teeth and NAFLD diagnosed by ultrasonography were investigated in 24,470 people. A significant correlation was found between the number of missing teeth and a higher presence of NAFLD in men but not women [14]. In Japan, Furuta et al. reported a correlation between alanine transaminase (ALT) and the incidence of periodontal disease [11]. In addition, Yoneda et al. reported a higher detection rate of *P. gingivalis* in saliva by polymerase chain reaction (PCR) in patients with biopsy-proven NAFLD and NASH than in healthy individuals [12]. Furthermore, Ahmed et al. reported that the combination of metabolic syndrome and a high ALT level was associated with periodontal pocket depth [15], and Morita et al. reported that periodontal disease was associated with the serum γGTP level (Table 1) [16]. 

Periodontitis, identified by Probing pocket depth ≥ 4 mm, was reported to be a risk factor for NAFLD, diagnosed by ultrasound or hepatic stiffness in a cross-sectional study of a Japanese oral health check population (*n* = 887 without NAFLD, *n* = 339 with NAFLD) [17], and correlated with liver stiffness in patients with NAFLD and advanced fibrosis [18].

In an epidemiological study of 5421 individuals with oral status and fatty liver in the National Health and Nutrition Examination Survey, adults with fewer than 20 remaining teeth, moderate to severe periodontitis, and untreated caries were each 1.5 times more likely to have NAFLD than their counterparts. The fibrosis index and periodontal disease were also reported to be significantly associated with a high odds ratio (OR) (OR = 3.1 95% confidence interval [CI]: 2.31–4.17) [19]. The serum level of the γGTP enzyme was also significantly higher in NAFLD subjects than their counterparts without NAFLD [17].

## 3. Pathophysiology Linking NAFLD/NASH and Periodontal Disease

*P. gingivalis*, closely related to periodontal disease, has been reported to be involved in the development of NAFLD and NASH. The periodontopathogenic bacterium *P. gingivalis* enters the bloodstream through the periodontitis lesions, and the pathogenic bacteria, their products, and inflammatory cytokines are transported throughout the body and reach the liver; Furusho et al. reported the presence of *P. gingivalis* in the liver tissue from the patients with NASH by immunohistochemical staining, and the presence of this organism in the liver is associated with hepatic fibrosis [20]. Furthermore, this report describes that when *P. gingivalis* infects mice with diet-induced fatty liver through the pulp chamber, the serum LPS concentration increases, and further liver steatosis progresses [20]. In other experiments using a mouse model of fatty liver, the intravenous administration of *P. gingivalis* and intraoral administration of *P. gingivalis* caused exacerbation of obesity, insulin resistance, and steatohepatitis [12,21]. It is mainly due to the endotoxin produced by *P. gingivalis*. The leading cause is thought to be the endotoxin lipopolysaccharide (LPS) produced by *P. gingivalis* and the innate immune system response via the TLR2/TLR4 pathway [20]. In vitro studies have shown that stimulation of the cell line HepG2 isolated from hepatocellular carcinoma with LPS from *P. gingivalis* resulted in an increase in MyF88, one of the adapter molecules that mediate both TLR2 and TLR4 signaling pathways, and an increase in proinflammatory cytokines. In this report, LPS from *P. gingivalis* contributes to intracellular lipid accumulation and inflammatory responses in HepG2 cells via activation of nuclear factor-κB and JNK signaling pathways [22]. Conversely, it has also been reported that lipid droplets in the liver affect *P. gingivalis*. A study using oleate-induced HepG2 cells reported that fat droplets in the liver increase intracellular *P. gingivalis* and that fat droplets affect the removal of *P. gingivalis* by altering the autophagy mechanism [23]. In a study examining the effects of *P. gingivalis*-derived LPS on immortalized human fetal hepatocytes (Hc3716-hTERT) induced to steatosis by palmitic acid, the expression of TLR2, one of the *P. gingivalis*-derived LPS receptors, was upregulated and mRNA levels of inflammasomes (NLRp 3, Casp-1) and proinflammatory cytokine were increased [20].

In a human study, *P. gingivalis* was detected by PCR in the saliva of 60, 48, and 102 healthy individuals with NAFL and NASH, respectively, and the positive rates were 21.7%, 35.4%, and 50.2%, respectively. Additionally, the frequencies of types II, Ib, and IV, which are highly pathogenic strains of *FimA*, were 50%, 30%, and 14.3%, respectively [12]. Serum immunoglobulin (Ig)-G antibody titers against *FimA* measured by enzyme-linked immunoassay were compared with histological findings in 200 cases of biopsy-proven NAFLD, and it was reported that the possession of *FimA* type IV was significantly correlated with advanced hepatic fibrosis in multivariate analysis [24]. Furthermore, in a cross-sectional study of 164 patients with NAFLD, *P. gingivalis* positivity in saliva (*P. gingivalis* ratio > 0.01%) was significantly correlated with liver stiffness assessed by magnetic resonance elastography (MRE) (*p* < 0.0001). Additionally, patients with NAFLD who were considered to have significant liver fibrosis according to Liver stiffness measurements by MRE had significantly elevated endotoxin activity in the blood. Note that in this study, NAFLD patients with more than 10 periodontal pockets ≥ 4 mm deep had significantly higher liver stiffness in both vibration-controlled transient elastography and MRE [18].

In addition to *P. gingivalis*, an association with *Aggregatibacter actinomycetemcomitans (A. actinomycetemcomitans)* has also been reported as a periodontopathogenic bacterium. Komazaki et al. reported that anti-A *A. actinomycetemcomitans* antibody levels in 52 patients with NAFLD were associated with visceral fat, the degree of insulin resistance and hepatic steatosis measured by computed tomography. Furthermore, administration of *A. actinomycetemcomitans* to mice resulted in glucose intolerance and insulin resistance, and administration of this organism to a mouse model of fatty liver exacerbated liver steatosis. Additionally, in mice treated with *A. actinomycetemcomitans*, 16rs RNA sequencing revealed that the gut microbiota is altered. Metagenomic prediction in the gut microbiota results in the up-regulation of fatty acid biosynthesis and down-regulation of fatty acid degradation. The study concluded that *A. actinomycetemcomitans* infection exacerbates fatty liver via changes in gut microbiota, glucose metabolism, and lipid metabolism [25]. 

**Figure 1:**
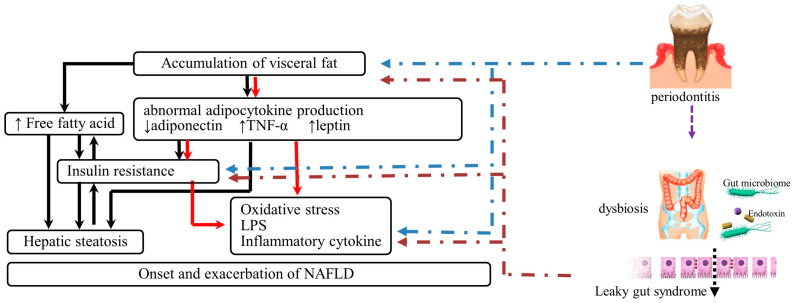
Pathogenic Mechanisms Linking Periodontal Disease and NAFLD. NAFLD, nonalcoholic fatty liver disease; TNF, tumour necrosis factor; LPS, lipopolysaccharide; ↑, increase in blood concentration; ↓, decrease in blood concentration.

Many reports support the association between NAFLD and periodontal disease, but since both diseases are closely related to metabolic functions, many factors are considered to be mutually involved (Figure 1).

## 4. Periodontal Disease and Oral–Gut–Liver Axis

Recently, new molecular biological techniques have revealed that intestinal bacteria are involved in the pathogenesis and progression of various diseases. The liver is the first target organ for bacteria, endotoxins, and metabolites that invade the portal vein from the intestinal tract. The effects of intestinal wall barrier dysfunction (leaky gut syndrome) [26,27,28], qualitative and quantitative abnormalities of intestinal bacteria, and increased endogenous alcohol are considered exacerbating factors in various liver diseases. Recently, an increasing number of reports have suggested that changes in the oral microbiota may affect the intestinal microbiota. Analysis of the intestinal microbiota of patients with liver cirrhosis revealed a decrease in species diversity. However, a high frequency of bacteria of oral origin (four species of *Streptococcus* and six species of *Veillonella*) correlated with disease severity, and the concept of an oral–gut–liver axis was proposed [29]. Humans produce 1–1.5 L of saliva daily, which contains 8.3 × 10^6^/mL of *P. gingivalis* in patients with moderate periodontitis, and it was reported that the intraoral administration of experimental *P. gingivalis* [21,30] and intravenous *P. gingivalis*-derived LPS [31] altered the intestinal microflora and microbiota.

Additionally, the oral administration of *P. gingivalis* causes disruption to the intestinal wall barrier function (leaky gut syndrome) [21,28]. Recently, Yamazaki et al. reported that the oral administration of *Prevotella intermedia* or *P. gingivalis* caused significantly more severe NAFLD in mice. Furthermore, the metagenomic analysis revealed that administering these oral bacteria increased the expression of genes involved in synthesising aromatic amino acids in the intestinal microflora, impaired intestinal barrier function, and caused endotoxinemia. In particular, the administration of *P. gingivalis* affected the expression of genes involved in the development and progression of NAFLD, such as those involved in the expression of the genes related to the endoplasmic reticulum stress, circadian rhythm, fibrosis, and tumorigenesis. In addition, it has been suggested that gingival pathogens swallowed with saliva may alter the composition and function of intestinal bacteria and adversely affect liver function by increasing intestinal permeability, which may be involved in the onset and progression of NAFLD [32] (Figure 1).

In an examination of intestinal bacteria, obesity-associated hyperleptinemia activated STAT3 signaling in Kupffer cells, which induced an excessive inflammatory response of the liver to LPS via increased expression of CD14, a co-receptor for TLR4, leading to the development of NASH pathogenesis [33]. 

## 5. Is Periodontal Therapy Useful as a Treatment for NAFLD?

There is still no established pharmacotherapy for NAFLD/NASH [34,35,36]. Currently, drug treatment in accordance with each factor of the metabolic syndrome associated with NAFLD/NASH is recommended. Although there are few reports on whether the periodontal intervention improves NAFLD pathophysiology, non-surgical periodontal treatment in 10 patients with NAFLD was reported to improve liver function test results, such as aspartate aminotransferase (ALT) and AST after 3 months [12], suggesting that periodontal treatment may be a helpful, supportive therapy in the management of NAFLD. Kamata et al. randomized 40 patients to a scaling and root planning (SRP) or tooth brushing group to evaluate the efficacy of periodontal treatment in patients with NAFLD. The SRP group showed significantly lower ALT levels and *P. gingivalis* IgG antibody titers than the toothbrushing group [37]. However, a large-scale, multicenter study is needed to verify the efficacy of periodontal therapy in patients with NAFLD. 

## 6. Relationship between Periodontal Disease and Atherosclerosis

In 2015, Angulo et al. reported that in a histological study of 619 patients with NAFLD/NASH observed for 12.6 years, 33.2% of patients died or underwent liver transplantation. Cardiovascular disease was the most common cause of death (38.3%)—more cardiovascular disease-related deaths than liver-related deaths, such as cirrhosis or hepatocellular carcinoma [38]. Similarly, in a European study of 646 NAFLD/NASH patients with histology from 2017 and a mean follow-up of 20 years, 33.1% of patients died; the most common cause of death was cardiovascular events (36.9%), followed by extrahepatic malignancies (25.7%), and liver-related death was fourth at 7.9% [39]. In NAFLD disease, liver fibrosis progression is strongly associated with liver disease and causes of death, including cardiovascular disease. Therefore, patients with NAFLD require appropriate evaluation for cardiovascular disease [36]. Periodontal disease has been reported to increase the incidence of atherosclerotic cardiovascular disease and mortality. A meta-analysis revealed that periodontal disease has a statistically significant association with atherosclerotic cardiovascular disease [40,41]. Elevated levels of inflammatory markers such as CRP, IL-6, IL-1, IL-8, and TNF-α in the blood of patients with periodontitis are thought to be associated with atherosclerotic lesion formation. In an analysis of 5297 patients with periodontal disease, patients with poor response to periodontal therapy reported a significantly increased risk of developing cardiovascular disease [42]. Systematic review and meta-analyses showed that periodontal treatment improves endothelial function and reduces biomarkers of atherosclerotic disease in those already suffering from cardiovascular disease and/or diabetes [43,44]. Because atherosclerosis also plays a role in the prognosis of patients with NAFLD, future studies on the relationship between periodontal disease and atherosclerosis are warranted. 

## 7. Relationship between Periodontal Disease and Diabetes Mellitus

Follow-up studies of patients with severe periodontitis have reported an increased risk of new onset of diabetes, worsening of glycated hemoglobin A1c (HbA1c), a marker of glucose control, and increased frequency of diabetic complications. Multiple mechanisms have been postulated to exacerbate periodontal disease in diabetic patients. First, dehydration due to high blood glucose causes dryness of the oral cavity and a decrease in self-cleansing by saliva. Second, leukocyte migration, phagocytosis, and bactericidal activity are reduced by hyperglycemia, resulting in decreased resistance to periodontopathogenic bacteria. Lastly, AGEs (advanced glycation end products), which are formed when excess glucose in the blood binds to proteins, alter the function of type I collagen and laminin, which are important substrate molecules of periodontal tissue. Inflammatory cytokines have also been reported to play a role in the development of diabetes. Tumour necrosis factor (TNF)-α is the best-known factor that inhibits insulin signalling. Although TNF-α levels are elevated in the lesions of periodontally ill patients, there is no agreement on whether they are also elevated in the blood and induce insulin resistance [45]. However, numerous studies have reported elevated levels of the inflammatory cytokine interleukin (IL)-6 in the blood of patients with periodontal disease [46]. In addition, IL-6 induces C-reactive protein (CRP) production in the liver [47]. IL-6 and CRP are involved in insulin resistance, and now these inflammatory factors are thought to be the cause of the adverse effect of periodontitis on glycemic control.

It has been shown that the severity of periodontal disease is significantly higher in diabetic patients [48], and diabetes play an important role because of the high association between periodontal disease and NAFLD. Furthermore, longitudinal studies on the progression of periodontal disease in patients with type 1 diabetes have revealed that periodontal disease progresses more frequently in the poorly glycemic-controlled group than in the well glycemic-controlled group [49,50] and that periodontal disease recurs more frequently after periodontal treatment [51]. 

In type 2 diabetes, a report based on a large epidemiological study in the Pima Tribe of the United States was made in 1990, showing that the incidence of periodontal disease in diabetic patients was 2.6 times higher than in non-diabetic patients [52]. The Third National Nutrition Examination Survey (NHANES III) reported that the odds ratio of severe periodontal disease in patients with type 2 diabetes was 2.90 times higher for HbA1c ≥ 9.0% and 1.56 times higher for HbA1c < 9.0% [53]. A German cohort study reported that diabetic patients with an HbA1c of 7.0% or higher had a significantly higher risk of periodontal disease progression and tooth loss [54]. A large 20-year cohort study of US men reported that type 2 diabetes increased the incidence of periodontal disease by 29% and tooth loss by 9% per year [55].

It is well known that hyperglycemia decreases immune function and alters the body’s response to inflammation and that diabetes increases the incidence and risk of progression of periodontal disease, which is an inflammatory disease. Periodontitis causes plaque accumulation and the formation of periodontal pockets with numerous micro-ulcers under the gingival margin. Inflammatory reactions mediated by periodontal pockets cause local alveolar bone resorption and affect the whole body as a minor chronic inflammation. The mechanism of inflammation is thought to include increased insulin resistance or decreased insulin sensitivity via inflammatory cytokines, which may worsen glycemic control and localized effects of AGEs on periodontal tissues [56]. Periodontal disease may also cause abnormal glucose tolerance. The high risk of glucose intolerance due to periodontal disease has been demonstrated in an NHANES III study [57] and the Hisayama Study from Japan [58]. In 2013, the joint consensus of the American Academy of Periodontology and the European Academy of Periodontology stated that mild to moderate periodontitis increases the risk of progression to diabetes and severe periodontitis worsens glycemic control [59]. In a cross-sectional study of the Japanese oral health examination population, HbA1c was significantly higher in participants with probing pocket depth ≥ 6 mm (*n* = 211) compared to participants with probing pocket depth ≤ 3 mm (*n* = 285) or 4–5 mm (*n* = 730) [17].

The systematic review of the European Academy of Periodontology and the International Diabetes Federation, which updated the same report by adding references in 2018, also stated that HbA1c and worse glycemic control by fasting blood glucose and oral glucose tolerance test were clearly associated with the presence of periodontal disease [60]. A recent meta-analysis showed that the mean improvement in the HbA1c level after 3 months of periodontal treatment was 0.4% [61]. The effect of periodontal disease on diabetes has been studied not only in terms of glycemic control but also in terms of the endpoint of the development of complications, and cohort studies have shown that diabetic patients with severe periodontitis have an increased incidence of diabetic nephropathy [62] and mortality due to ischemic heart disease [63].

## 8. Relationship between Periodontal Disease and Cancer

Periodontal disease has been reported to be associated with a risk of malignancy [64,65]. The abundance and richness of the intestinal microbiota are increased in patients with colorectal cancer compared to healthy individuals. One of the factors is thought to be the ectopic colonization of bacteria derived from the oral cavity. In particular, *Fusobacterium nucleatum* (*F. nucleatum*) of oral origin, which is involved in the development of periodontal disease, promotes the proliferation and migration of colon cancer cells and is strongly associated with the development of colon cancer (4). In patients with colorectal cancer, the same strains of *F. nucleatum* were detected in the oral cavity and tumors. This report suggests that *F. nucleatum* of oral origin is involved in the development of colorectal cancer and that prevention of colorectal cancer may be possible by targeting oral bacteria [66].

Regarding other cancer types, several cohort studies have noted a significantly increased risk of pancreatic cancer in patients with periodontal disease compared to healthy individuals. For example, a meta-analysis by Michaud et al. reported five cohort studies on periodontal disease and pancreatic cancer, all of which showed a more than 50% increased risk of pancreatic cancer in the presence of periodontal disease. The hazard risk for pancreatic cancer is reported to be 1.54–4.56, which is statistically significant in three of the five cohort studies. [65,67]. Two cohort studies have also reported an association between periodontal disease and the risk of developing breast cancer. According to these studies, the risk of developing breast cancer is significantly 1.14 to 1.36 times higher in patients with periodontal disease than in healthy individuals [68,69].

Additionally, it was reported that *P. gingivalis*, a major causative agent of periodontal disease, was increased in the esophageal flora of esophageal cancer patients, and that *P. gingivalis* was positively correlated with several clinicopathological features of esophageal cancer, such as differentiation status, metastasis, and overall survival [70,71]. Thus, oral bacteria and periodontal disease have been reported to be involved in malignancy in multiple carcinomas. With respect to hepatocellular carcinoma (HCC), periodontal disease has been reported to correlate with the HCC stage [72]. However, there are few reports on the relationship between periodontal disease and HCC, and further research in this area is warranted.

## 9. Prognosis of NAFLD/NASH Associated with Periodontal Disease

Prospective cohort studies on the association between periodontal disease and NAFLD have also been published. In a 5-year follow-up study of 341 patients with NAFLD, Kuroe et al. showed that the progression of liver fibrosis was strongly associated with the clinical attachment level (CAL), which indicates the degree of destruction of periodontal tissue in patients with obesity (OR = 2.87, 95% CI: 1.23–6.69) [73]. In a German study of 2623 individuals over a median of 7.7 years, 605 cases of NAFLD occurred (32.5 cases per 1000 person-years); the risk of developing new NAFLD was 1.28 times higher in those with CAL (≤30%) than in those without CAL (≥30%), and that was 1.60 times more frequent in extensive CAL-periodontitis [74]. In addition, it has been suggested that periodontitis is associated with the rate of fibrosis development in the liver [75]. A 13-year follow-up study of 1801 patients with NAFLD in Finland found that severe periodontitis significantly increased the occurrence of liver-related events (hospitalization for liver disease, liver disease-related fat, and diagnosis of liver cancer) (HR = 6.94, 95% CI: 1.43–33.6) in patients with severe periodontitis [76].

These reports suggest that periodontitis may contribute not only to the development of NAFLD but also to its severity.

## 10. Conclusions

Forty years have passed since the concepts of NAFLD, and NASH were first proposed; however, no effective prevention or treatment has been established. The pathogenesis of NAFLD/NASH is not limited to liver-related diseases. Still, it has been reported to be associated with various systemic diseases and an increasing number of causes of death. Studies on periodontal disease and NAFLD/NASH are expected to elucidate the bi-directional relationship between periodontal disease and NAFLD/NASH, as both are associated with lifestyle-related diseases. Recently, there has been a series of reports suggesting a relationship between oral and intestinal microflora. Changes in the intestinal microbiota have been shown to be a risk factor for periodontal diseases, such as atherosclerosis, diabetes, rheumatoid arthritis, nonalcoholic fatty liver disease, and obesity. Future integrated analysis of the effects of *P. gingivalis* and other oral bacteria, identification of fluctuating intestinal bacteria and their relationship to pathogenesis, and changes in metabolites and their effects on the immune system should lead to clarification of the pathogenesis of periodontal disease and NAFLD and the development of new treatment methods.

## Figures and Tables

**Table 1:** Cross-sectional studies of periodontitis and NAFLD.

Author	Country	Year Published	Number of Participants	Result
Kim et al. [13]	Korea	2020	4272	The fatty liver index was associated with periodontal disease.
Qiao et al. [14]	China	2018	24,470	The number of missing teeth was associated with a higher presence of NAFLD in men.
Furuta et al. [11]	Japan	2010	2225	High ALT levels were associated with periodontal disease.
Yoneda et al. [12]	Japan	2012	150	There was a higher detection rate of *P. gingivalis* in saliva by PCR in patients with biopsy-proven NAFLD and NASH than in healthy participants.
Ahmed et al. [15]	Japan	2017	5683	A combination of metabolic syndrome and a high ALT level was associated with the periodontal pocket depth.
Morita et al. [16]	Japan	2014	1510	Periodontal disease was associated with the serum γGTP level.

ALT, alanine transaminase; PCR, polymerase chain reaction; *P. gingivalis*, *Porphyromonas gingivalis*; NAFLD, nonalcoholic fatty liver disease; NASH, nonalcoholic steatohepatitis.

## Data Availability

Not applicable.
